# Assessing the effect of obesity-related traits on multiple myeloma using a Mendelian randomisation approach

**DOI:** 10.1038/bcj.2017.48

**Published:** 2017-06-16

**Authors:** M Went, A Sud, P J Law, D C Johnson, N Weinhold, A Försti, M van Duin, J S Mitchell, B Chen, R Kuiper, O W Stephens, U Bertsch, C Campo, H Einsele, W M Gregory, M Henrion, J Hillengass, P Hoffmann, G H Jackson, O Lenive, J Nickel, M M Nöthen, M I da Silva Filho, H Thomsen, B A Walker, A Broyl, F E Davies, C Langer, M Hansson, M Kaiser, P Sonneveld, H Goldschmidt, K Hemminki, B Nilsson, G J Morgan, R S Houlston

**Affiliations:** 1 Division of Genetics and Epidemiology, The Institute of Cancer Research, London, UK; 2 Division of Molecular Pathology, The Institute of Cancer Research, London, UK; 3 Myeloma Institute for Research and Therapy, University of Arkansas for Medical Sciences, Little Rock, AR, USA; 4 Department of Internal Medicine V, University of Heidelberg, Heidelberg, Germany; 5 Molecular Genetic Epidemiology, German Cancer Research Center, Heidelberg, Germany; 6 Center for Primary Health Care Research, Lund University, Malmo, Sweden; 7 Department of Hematology, Erasmus MC Cancer Institute, Rotterdam, The Netherlands; 8 National Center for Tumor Diseases, Heidelberg, Germany; 9 Department of Internal Medicine II, Division of Hematology and Medical Oncology, University Hospital Würzburg, Würzburg, Germany; 10 Clinical Trials Research Unit, Leeds Institute of Clinical Trials Research, University of Leeds, Leeds, UK; 11 Institute of Human Genetics, University of Bonn, Bonn, Germany; 12 Division of Medical Genetics, Department of Biomedicine, University of Basel, Basel, Switzerland; 13 Royal Victoria Infirmary, Newcastle upon Tyne, Newcastle, UK; 14 Department of Genomics, Life & Brain Center, University of Bonn, Bonn, Germany; 15 Department of Internal Medicine III, University of Ulm, Ulm, Germany; 16 Division of Hematology and Transfusion Medicine, Department of Laboratory Medicine, Lund University, Lund, Sweden; 17 Hematology Clinic, Skåne University Hospital, Lund, Sweden; 18 Clinical Immunology and Transfusion Medicine, Laboratory Medicine, Office of Medical Services, Lund, Sweden; 19 Broad Institute, 7 Cambridge Center, Cambridge, MA, USA

Multiple myeloma (MM) accounts for around 15% of new cases and 20% of deaths amongst patients diagnosed with haematological malignancy. To date, few risk factors have been robustly confirmed for MM, these include increasing age, male sex, black race and a family history of MM.^1^

High body mass index (BMI) has been reported to be associated with an increased risk of MM in several observational studies, though questions remain regarding the aetiological relevance, including the distribution of body fat.^2^ In addition to being a potential risk factor for MM, some, but not all, studies have suggested an association between BMI and prognosis.^1, 3^ A recent study has suggested the relationship between BMI and MM may be through reduced levels of plasma adiponectin, the inflammatory mediator secreted by adipocytes. The association was, however, confined to obese individuals providing an argument against a direct causal link.^4^ Findings such as these, alongside the conflicting results of previous studies into adiposity traits, highlight the limitations of observational studies. Importantly, such studies do not establish a causal relationship, as they cannot fully eliminate the influence of confounding factors. Moreover, in the context of prognostication, many studies have not explicitly addressed the issue of reverse causation.^5^

Mendelian randomisation (MR) provides an attractive alternative to the traditional epidemiological study for examining relationships between exposure and disease. MR makes use of allelic variants, which are randomly assigned during meiosis and are robustly associated with traits of interest, as instrumental variables (IVs) to infer whether associations between exposure and disease are causal. The use of these genetically defined IVs as proxies of modifiable exposure, avoids confounding by environmental factors, can be reflective of life-long exposure and circumvents reverse causality.

Genome-wide association studies (GWAS) have identified single-nucleotide polymorphisms (SNPs) at multiple independent loci significantly associated with BMI, childhood obesity (CHO) and plasma levels of adiponectin.^6, 7, 8^ Here we have sought to establish a causal association between adiposity traits and MM by performing a MR analysis using SNPs associated with body mass index (BMI), hip circumference adjusted for BMI (HipadjBMI), waist circumference adjusted for BMI (WCadjBMI), waist-to-hip ratio adjusted for BMI (WHRadjBMI), CHO and plasma adiponectin levels as IVs.

We constructed genetic risk scores (GRS), to investigate the relationship of adiposity and plasma levels of adiponectin with MM risk, using the data from five reported MM GWASs, comprising 6 839 cases and 22 221 controls.^9^ We performed two-stage MR analysis to assess the association between each adiposity trait and MM using summary statistics from the MM GWAS, and the published effect size of the adiposity trait. As per Burgess *et al.*,^10^ a fixed-effects model was used to calculate combined ratio estimates, β, for the effect of each trait on MM risk. Results are summarised in Table 1.

Following calculation of combined ratio estimates, β, in three of the five cohorts, a positive association was shown between one s.d. in BMI (kg /m^2^) and MM risk, with the UK-GWAS series being nominally significant (Figure 1a). Meta-analysis of the data from all five cohorts did not, however, provide evidence for a causal association (odds ratio (OR)=1.17, 95% confidence intervals (CIs): 0.92–1.49, *P*=0.19). Similarly, we found no association between the other anthropometric traits, which report on central obesity and MM risk, specifically—HipadjBMI, WCadjBMI, WHRadjBMI, which had respective ORs of 0.77 (95% CI: 0.42–1.41), 0.62 (95% CI: 0.37–1.02) and 0.82 (95% CI: 0.57–1.19) (Figures 1b–d). We also found no support for a relationship between CHO and MM risk (OR=0.98, 95% CI: 0.87–1.10; Figure 1e). We then evaluated the impact of plasma levels of adiponectin on the risk of developing MM, again observing no association (OR=1.04, 95% CI: 0.64–1.72; Figure 1f).

Linkage of the survival data to genotypes on three of the series allowed the relationship between the aforementioned adiposity-related traits and patient outcome to be examined through MR. In meta- analyses of these data, no association between any of the traits and either overall survival (OS) or progression-free survival (PFS) was shown.

The results from our study contrast with some observational epidemiological studies, which have shown a positive association between adiposity and MM risk and mortality.^1, 11^ A recent meta-analysis of studies found a modest, but significant association between BMI and risk in prospective cohorts.^11^ Reported relative risks for MM associated with obesity from these studies had OR 95% CIs of 1.08–1.35. Over this range of effect, we had 16–97% study power to demonstrate an association. Hence, we cannot exclude the possibility that the null results we observed were simply a consequence of limited study power if the true effect is marginal.

Obesity is a well-established risk factor for a number of other solid cancers.^12^ By inference, it is likely *a priori* that obesity will also increase MM risk. An elevated level of insulin-like growth factor 1, associated with chronic hyperinsulinemia, stimulates cell proliferation and inhibits apoptosis, therefore providing a biological basis for obesity having a generic effect on cancer risk.^13^ In the case of MM, the insulin receptor is overexpressed in MM cells and is increased throughout normal plasma cell differentiation.^14^ These observations would therefore imply a causative effect of obesity and ensuing hyperinsulinemia on plasma and myeloma cell growth; a corollary of this may be increased risk of MM and adverse patient outcome.

Our MR analysis does not suffer from the influence of recall bias and confounding that can affect observational studies.^5^ Nevertheless, a central assumption in MR is that the variants used as IVs are associated with the exposure being investigated. To ensure this was the case, we only used SNPs associated with adiposity-related traits at genome-wide significance from GWAS. Furthermore, we only used the data from individuals of European descent to limit bias from population stratification. An additional assumption is that the variants are associated with MM only through the exposure and are not confounded by pleiotropy. We assessed the impact of possible pleiotropism on MR estimates using both inverse variance weighted (IVW) and MR-Egger regression tests as per Bowden *et al.*^15^ Neither test provided evidence for pleiotropy, with respective *P*-values of 0.2 and 0.71 for BMI, 0.77 and 0.65 for CHO, 0.56 and 0.52 for HipadjBMI, 0.13 and 0.66 for WCadjBMI, 0.4 and 0.79 for WHRadjBMI, and 0.87 and 0.2 for adiponectin plasma levels. While we found no evidence that the SNPs violated this IV assumption, this does not exclude confounding by as yet unidentified confounders.

In conclusion, high BMI is a plausible risk factor for MM; however, observational studies so far have provided varied and conflicting results, likely attributed to confounding and reverse causation. Our MR study, which uses IVs to avoid such confounding factors, provides no evidence of BMI or other tested adiposity traits, influencing MM risk or survival. To robustly establish a causal relationship through MR-based analyses, and thus avoid confounding, far larger datasets will be required than the one we have analysed. Such studies should be possible in the future with ongoing GWASs of MM currently being undertaken.

## Figures and Tables

**Figure 1:**
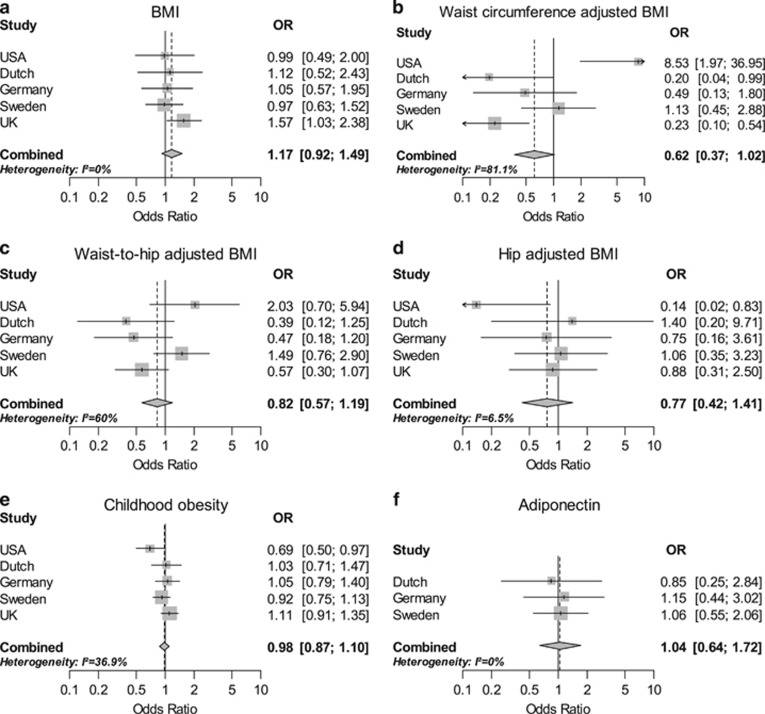
Meta-analysis odds ratios (OR) for multiple myeloma per unit increase in genetic risk score (s.d. trait) for each adiposity trait. Shaded boxes denote odds ratio for individual cohorts with areas proportional to the inverse variance weight of the estimate. Horizontal lines represent 95% confidence intervals (CIs) for individual cohorts. Shaded diamond represents summary ORs, computed under a fixed-effects models and diamond width gives 95% CIs. Solid vertical line represents null hypothesis (OR=1) and dashed vertical line indicates OR from meta-analysis. (**a**) BMI. (**b**) Waist circumference adjusted for BMI. (**c**) Waist-to-hip ratio adjusted for BMI. (**d**) Hip circumference adjusted for BMI. (**e**) Childhood obesity. (**f**) Adiponectin levels.

**Table 1:** Results of associations of multiple myeloma (MM) risk with adiposity traits using Mendelian randomisation

*Trait*	*Odds ratio*	*95% CI*	P*-value*
BMI	1.17	0.92;1.49	0.19
CHO	0.98	0.87;1.10	0.71
WHRadjBMI	0.82	0.57;1.19	0.29
WCadjBMI	0.62	0.37;1.02	0.06
HipadjBMI	0.77	0.42;1.41	0.39
Adiponectin^a^	1.04	0.64;1.72	0.86

Abbreviations: BMI, body mass index; CHO, childhood obesity; HipadjBMI, hip circumference adjusted for BMI; WCadjBMI, waist circumference adjusted for BMI; WHRadjBMI, waist-to-hip ratio adjusted for BMI.

Odds ratio, 95% confidence intervals (CIs) and and *P*-values from meta-analysis of all cohorts, which demonstrates no significant causative effect of traits on MM risk.

a Meta-analysis for adiponectin was conducted with 3 cohorts as no SNPs were present in UK and USA data sets.

## References

[bib1] Teras LR, Kitahara CM, Birmann BM, Hartge PA, Wang SS, Robien K et al. Body size and multiple myeloma mortality: a pooled analysis of 20 prospective studies. Br J Haematol 2014; 166: 667–676. 24861847 10.1111/bjh.12935 PMC4134758

[bib2] Carson KR, Bates ML, Tomasson MH. The skinny on obesity and plasma cell myeloma: a review of the literature. Bone Marrow Transplant 2014; 49: 1009–1015. 24820216 10.1038/bmt.2014.71

[bib3] Beason TS, Chang SH, Sanfilippo KM, Luo S, Colditz GA, Vij R et al. Influence of body mass index on survival in veterans with multiple myeloma. Oncologist 2013; 18: 1074–1079. 24048366 10.1634/theoncologist.2013-0015 PMC3805147

[bib4] Hofmann JN, Birmann BM, Teras LR, Pfeiffer RM, Wang Y, Albanes D et al. Low levels of circulating adiponectin are associated with multiple myeloma risk in overweight and obese individuals. Cancer Res 2016; 76: 1935–1941. 26921332 10.1158/0008-5472.CAN-15-2406 PMC4878138

[bib5] Berrigan D, Troiano RP, Graubard BI. BMI and mortality: the limits of epidemiological evidence. Lancet 2016; 388: 734–736. 27423263 10.1016/S0140-6736(16)30949-7 PMC5508818

[bib6] Dastani Z, Hivert MF, Timpson N, Perry JR, Yuan X, Scott RA et al. Novel loci for adiponectin levels and their influence on type 2 diabetes and metabolic traits: a multi-ethnic meta-analysis of 45,891 individuals. PLoS Genet 2012; 8: e1002607. 22479202 10.1371/journal.pgen.1002607 PMC3315470

[bib7] Locke AE, Kahali B, Berndt SI, Justice AE, Pers TH, Day FR et al. Genetic studies of body mass index yield new insights for obesity biology. Nature 2015; 518: 197–206. 25673413 10.1038/nature14177 PMC4382211

[bib8] Shungin D, Winkler TW, Croteau-Chonka DC, Ferreira T, Locke AE, Magi R et al. New genetic loci link adipose and insulin biology to body fat distribution. Nature 2015; 518: 187–196. 25673412 10.1038/nature14132 PMC4338562

[bib9] Mitchell JS, Li N, Weinhold N, Forsti A, Ali M, van Duin M et al. Genome-wide association study identifies multiple susceptibility loci for multiple myeloma. Nat Commun 2016; 7: 12050. 27363682 10.1038/ncomms12050 PMC4932178

[bib10] Burgess S, Butterworth A, Thompson SG. Mendelian randomization analysis with multiple genetic variants using summarized data. Genet Epidemiol 2013; 37: 658–665. 24114802 10.1002/gepi.21758 PMC4377079

[bib11] Wallin A, Larsson SC. Body mass index and risk of multiple myeloma: a meta-analysis of prospective studies. Eur J Cancer 2011; 47: 1606–1615. 21354783 10.1016/j.ejca.2011.01.020

[bib12] De Pergola G, Silvestris F. Obesity as a major risk factor for cancer. J Obes 2013; 2013: 291546. 24073332 10.1155/2013/291546 PMC3773450

[bib13] Kumari N, Dwarakanath BS, Das A, Bhatt AN. Role of interleukin-6 in cancer progression and therapeutic resistance. Tumour Biol 2016; 37: 11553–11572. 27260630 10.1007/s13277-016-5098-7

[bib14] Sprynski AC, Hose D, Kassambara A, Vincent L, Jourdan M, Rossi JF et al. Insulin is a potent myeloma cell growth factor through insulin/IGF-1 hybrid receptor activation. Leukemia 2010; 24: 1940–1950. 20844560 10.1038/leu.2010.192 PMC3141222

[bib15] Bowden J, Davey Smith G, Burgess S. Mendelian randomization with invalid instruments: effect estimation and bias detection through Egger regression. Int J Epidemiol 2015; 44: 512–525. 26050253 10.1093/ije/dyv080 PMC4469799

